# Student-Reported Attitudes during an Interprofessional Palliative Care Learning Experience: Implications for Dual-Professional Identity, Interdisciplinary Bias, and Patient Outcomes

**DOI:** 10.1089/pmr.2020.0096

**Published:** 2020-12-11

**Authors:** Nassrine Noureddine, Darla K. Hagge, Pouria Kashkouli

**Affiliations:** ^1^ School of Nursing, California State University Sacramento, Sacramento, California, USA.; ^2^ Department of Communication Sciences and Disorders, California State University Sacramento, Sacramento, California, USA.; ^3^ Hospital Medicine and Palliative Care, University of California, Department of Internal Medicine, Davis Medical Center, Sacramento, California, USA.

**Keywords:** dual-professional identity, end of life, interdisciplinary bias, interprofessional education, multidisciplinary education, palliative care

## Abstract

***Background:*** The geriatric population in the United States is in need of palliative care (PC), yet it is not consistently established in the curriculum across health care training programs. There is a clarion call to reform the education of health care students using interprofessional education (IPE). The Joint Commission reported that communication errors represent two-thirds of the causes behind provider sentinel events in health care.

***Objective:*** The purpose of this study was to design, implement, and assess an IPE curriculum on PC to understand interprofessional student attitudes.

***Design/Setting:*** Three professors conducted a mixed-methods study at a California university involving an IPE PC event for 40 nursing and speech-language pathology students, and administered the Interprofessional Attitudes Survey (IPAS) and reflective questions.

***Results:*** Qualitative findings indicated that students increased their knowledge about PC and the purpose/value of IPE. Four out of the five IPAS subscales had positive outcomes: teamwork and roles/responsibilities, patient-centeredness, diversity/ethics, and community-centeredness. Interprofessional-biases subscale revealed that 33% of the participants reported biases toward students from other health care disciplines, and 35% reported that students from other health care disciplines held similar biases toward them. However, only 25% did not believe that the interdisciplinary biases interfered with patient outcomes.

***Conclusion:*** The study identified the existence of interprofessional biases and prejudices that may impede collaboration among health care professionals resulting in reduced health care outcomes. Faculty and health educators are encouraged to embed IPE into a multidisciplinary curriculum that dismantles preexisting interdisciplinary biases and stereotypes, and constructs dual-professional identity. IRB ID #904203-1

## Introduction

Interprofessional Collaborative Practice (IPCP) is a key element in the provision of holistic care among the multidisciplinary members of a palliative care (PC) team. IPCP, as an interprofessional collaborative service model, supports the development and maintenance of effective interprofessional relationships among all members of a health care team. Hence, PC can serve as a cutting-edge, collaborative service delivery model for a variety of health care-related disciplines while also dismantling traditional, long-standing silos that divide health care disciplines.

### Interprofessional education/IPCP

According to multiple national agencies in the United States, communication breakdowns between health care professionals are the primary reason for sentinel errors.^3–6^ In addition, the Joint Commission (JCAHO) reported that communication errors were the leading cause in almost every sentinel event between 2004 and 2014.^7^ As a result, JCAHO published the National Patient Safety Goals in an effort to improve communication interactions between health care professionals, including the recommendation that each health care facility should create a culture that encourages interprofessional teamwork.^8^ In addition, multiple agencies have recommended that health care professionals participate in continuing education programs to develop and improve interprofessional communication skills.^2–5^,^14^

Optimal health care outcomes can be achieved when a multidisciplinary team delivers comprehensive interprofessional services while working collaboratively with patients, families, and caregivers.^9^ It is crucial that health care students are prepared to work collaboratively across professions in today's dynamic health care environment.^9^ Many graduating health care professionals, however, are not collaborative-ready when they enter the workforce because of a paucity of formal interprofessional education (IPE) curricula in educational programs.^10^ IPE occurs when students from multiple disciplines are brought together to learn about, from, and with each other.^9^

### Accreditation agencies

In addition to the purposeful integration of IPCP in health care settings, accreditation agencies across health care disciplines in the United States have responded proactively to the call for a collaborative-ready health care workforce by mandating the inclusion of IPE across disciplines within academic curricula. Many health care-related training programs now require IPE, including but not limited to pharmacy, medicine, nursing, dentistry, physical therapy, social work, and speech-language pathology (SLP).^11^ Faculty are beginning to participate in specialized training to acquire the knowledge and skills necessary to design, implement, and assess literature-based IPE events and activities.^12^

### Competencies

PC has rapidly grown over the last 15 years and represents a significant paradigm shift in health care delivery, yet it represents a gap in nursing and speech-language-pathology curricula.^16^ The World Health Organization defines PC as an approach to health care services that improves the quality of life of patients and their families who face multiple problems typically associated with serious illness.^1,2^ PC is designed to prevent and relieve suffering by means of early identification, impeccable assessment, treatment of pain and other problems, including physical, psychosocial, and spiritual concerns across the life span.^2^

PC competencies are similar to the four IPE competencies, which include (1) values and ethics, (2) roles and responsibilities, (3) communication, and (4) teamwork.^13,14^ Due to the similarities between PC and IPE competencies, PC clinicians may be uniquely positioned to model IPCP to health care students. Moreover, there is a growing need for PC services as the percentage of our population older than 65 years continues to grow. The geriatric population is anticipated to represent 20% of the total U.S. population by 2030.^15,16^ The elderly typically present with multiple chronic conditions and are expected to consume 66% of the country's future health care budget.^17^ With the anticipated increase in the need for PC services, students must be prepared to work effectively with this population, to which interprofessional teamwork is the key. Collaborative care across disciplines is the gold standard of PC, and IPE is the best pedagogy to prepare a collaborative-ready health care workforce.^9^

### Purpose of study

The learning objectives of the designed IPE curriculum were as follows: (1) to explain the management of the different health care challenges encountered when dealing with end-of-life issues (e.g., pain, fatigue, nutrition, medications, grief and caregiver stress, the difference between Physician Orders for Life-Sustaining Treatment [POLST] vs. Advanced Directives)^18^; (2) to create a collaborative plan of care that is patient centered, and culturally and linguistically competent; (3) to discuss values/ethics for IPCP during end of life; (4) to identify roles and responsibilities for IPCP on a PC team; (5) to practice interprofessional communication across disciplines; and (6) to demonstrate interprofessional teamwork for optimal patient outcomes.

To meet the learning objectives, the authors determined that a case study of a patient with advanced-stage cancer with dysphagia would be a relevant clinical experience for nursing and SLP students. PC is within the scope of practice for nursing and SLP, yet it is rarely integrated into the curriculum for either discipline.

The purpose of this mixed-methods study was to design, implement, and assess a clinically relevant, interprofessional curriculum on PC that promotes the development of IPE competencies, in addition to measuring the self-reported interprofessional attitudes of participating health care students. The research question was: What is the impact of an IPE learning experience on the self-reported learning outcomes of nursing and SLP students?

## Methods

Based on the recommendations of the Institute of Medicine (IOM), a mixed-methods research design was implemented for this study.^5^ The quantitative section used a one-group post-test-only design, while the qualitative section included open-ended questions. A purposive convenience sample of nursing students in their last semester before licensure eligibility and SLP graduate students in their last academic year before licensure eligibility were recruited (*n* = 40). Institutional Review Board approval was obtained.

The three-hour IPE event was held at a Northern California University. The IPE PC curriculum was designed by an interuniversity, interprofessional team of two nursing professors, an SLP professor, and a medical professor who specializes in PC. The IPE activity included the following: (1) prereading assignments that were related to PC and represented three disciplinary perspectives; (2) didactic instruction; and (3) a team-based, unfolding case study.

The students were divided into interprofessional teams and completed an informed consent, a demographic survey, the Interprofessional Attitudes Survey (IPAS),^19^ and two reflective questions. According to the IOM recommendations for the evaluation of formal IPE interventions, the IPAS was identified as Level 2a, Modification in Attitudes and Perceptions, using Kirkpatrick's Expanded Outcomes Typology.^5,20^ See Table 1 for additional information regarding Kirkpatrick's typology. Descriptive analysis was used to analyze the quantitative data. The qualitative data were analyzed for emerging themes.

**Table 1.** Kirkpatrick's Expanded Outcomes Typology

Level	Description
Reaction	
Level 1	Students report view on the learning experience, including interprofessional experience
Learning	
Level 2a: Modification of attitudes/perceptions	Group changes in attitudes or perceptions
Learning	
Level 2b: Acquisition of knowledge/skills	Including knowledge and skills related to interprofessional collaboration
Behavior	
Level 3	Identifies students' transfer of IP learning to a practice setting and change in professional practice
Results	
Level 4a: Change in organizational practice	Broader changes in an organization and delivery of care
Results	
Level 4b: Benefits to patients or clients	Improvements in the health and/or well-being of patients/clients

Modified from Reeves et al.^20^

## Results

### Demographics

**FIG. 1.**
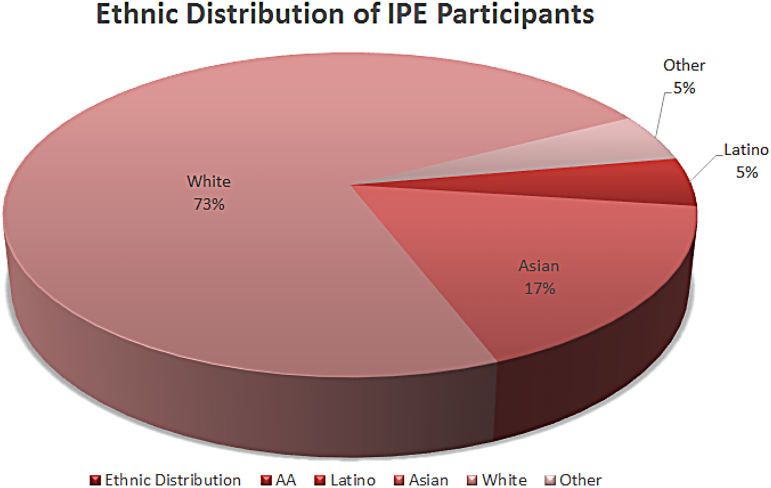
Ethnic distribution of interprofessional education participants.

A total of 40 students participated in the event. There were 70% nursing students (*n* = 28) and 30% SLP (*n* = 12). Overall, 82% of the students were female (*n* = 33) and 18% were male (*n* = 7). For ethnic distribution information, see Fig. 1.

### Quantitative results

The IPAS consists of five subscales: (1) teamwork, roles, and responsibilities, (2) patient-centeredness, (3) interprofessional biases, (4) diversity and ethics, and (5) community-centeredness. The survey results are discussed below.

#### Teamwork and responsibilities

Between 90% and 97% of the students self-reported a favorable response (i.e., agree or strongly agree) to the shared learning interprofessional event, indicating that they felt the experience: (1) made them a more effective health care team member, (2) improved their positive attitude about other professionals, (3) increased their ability to understand clinical problems, (4) provided insight into their own limitations, and (5) improved their communication ability with future patients and other professionals. When asked if they would value the opportunity to work on small interprofessional group projects with other students, 85% reported a willingness to participate with this kind of opportunity. Finally, 95% of participants reported a belief that it is necessary for health sciences students to learn together, and that their future patients would benefit if health care sciences students collaborated together to solve patient problems.

#### Patient centeredness

A total of 97% of the participants agreed that health care professionals need skills in interacting and cooperating with patients. They valued establishing trust with their patients, and they understood the importance of communicating compassion to patients. Ninety-five percent of the participants indicated that it is important to think about the patient as a person when providing treatment, and 97.5% of participants reported that it is important to understand the patient's perspective of the problem.

#### Interprofessional biases

Thirty-five percent of the students reported agreement or strong agreement that health care professional students from other health care disciplines have prejudices or make assumptions about them because of the discipline they are studying. Thirty-three percent of students reported agreement or strong agreement with having prejudices and making assumptions about health professionals from other disciplines. When asked if prejudices and assumptions about health professionals get in the way of delivery of health care, 75% of students agreed or strongly agreed with that statement.

#### Diversity and ethics

The majority (95%) reported that it is important for health care professionals to respect the unique cultures, values, roles/responsibilities, and expertise of other health professionals. A total of 97% of participants indicated that it is important for health care professionals to (1) provide excellent treatment to patients regardless of their background, (2) respect the dignity and privacy of patients, while maintaining confidentiality in the delivery of team-based care, and (3) understand how to communicate effectively across cultures.

#### Community centeredness

Ninety-seven percent of respondents agreed or strongly agreed that it is important to work with health care administrators and policy makers to improve delivery of health care, and 95% of respondents agreed or strongly agreed that it is important for health care professionals to work with legislators to develop laws, regulations, and policies that improve health care. A total of 97.5% of respondents agreed that it is important for health care professionals to advocate for the health of patients and communities. They need to remain focused on populations and communities, along with individual patients, to achieve effective health care delivery. Finally, 95% of respondents agreed or strongly agreed that it is important for health professionals to work with nonclinicians to deliver more effective health care.

### Qualitative results

Immediately following the three-hour IPE event, two open-ended prompts were presented to the participants to assess student learning. The first query was: *What did you find most helpful/effective about this IPE experience?* And the second prompt was: *Name and discuss three new things that you learned from this interprofessional learning experience*. The collected qualitative data were analyzed and resulted in the identification of two emerging themes, including *Understanding Hospice and Palliative Care* and *Understanding the Importance of IPEC domains* (Table 2). As previously stated, these emerging themes align with literature-based domains and include teamwork, collaboration, and roles and responsibilities.

**Table 2.** Qualitative Student Excerpts

Theme I: Understanding hospice and palliative care	Theme II: Importance of IPEC domains: teamwork, collaboration, and understanding roles and responsibilities
SLP: “Differentiating between hospice and palliative care”	SLP: “Finding out about the team members that need to be included in palliative care”
N: “The explanation of palliative care, and decision trees for end-of-life care”	N: “I enjoyed the presentation and getting to talk with students from the SLP program. It was good to see the scope of their knowledge and how it applies to those going through the end stages of death”
	N: “Collaboration with other professionals taught me the importance of working together when providing patient care”
	SLP: “The importance of working together because everyone has their different expertise”

IPEC, Interprofessional Education Collaborative; SLP, speech-language pathology.

## Discussion

### Interprofessional bias and prejudice

Based on the self-reported qualitative data (Table 2), the IPE learning experience contributed to participants' knowledge of PC, and increased their understanding of the clinical value of IPE and IPCP. The results from the quantitative data section, entitled Interprofessional Biases, which included self-reported participant biases, were noteworthy. As previously stated, more than a third (35%) of the participants agreed or strongly agreed that students from other health care professions had prejudices or made assumptions about them, while another third (37%) indicated that they were undecided. Yet, less than a third of the participants (28%) disagreed or strongly disagreed with this statement.

Thirty-three percent of the participants agreed or strongly agreed that they hold assumptions and biases about students from other professions, with 22% of the participants reporting that they were undecided, and more than 45% of the respondents reporting disagreement. When asked if prejudices and assumptions get in the way of the delivery of health care, the majority of the participants (75%) reported agreement or strong agreement, with only a few respondents (8%) reporting disagreement.

The participants reported biases or assumptions toward other health care students, and reported that other health care professionals held similar biases. This may be attributed to their inability to see beyond their own professional identities and recognize their role within the larger health care team. In short, the results of this study support the large body of literature indicating the need to break and dismantle the existing disciplinary silos in education training programs.^4–9,11,14,24^

The existence of the students' self-reported interprofessional bias and prejudice was surprising to the authors since this cadre of students had the opportunity to work together during multiple IPE events before participating in this study. This triggered the authors to investigate the literature for possible explanations behind students' self-reported bias and prejudice toward other disciplines. In the process, the authors discovered the concept of dual-professional identity (DPI) to explain the phenomenon.^24^ DPI is a new concept in IPE/IPCP.^24^

According to the principles of DPI, interprofessional bias and prejudice can be mitigated if students receive direct instruction during IPE clinical and/or didactic training events that target dismantling preexisting interprofessional biases and constructing DPI.^24^ DPI serves to expand the perceived “we” of an ingroup (e.g., nursing or SLP) by increasing the ingroup's inclusiveness between different groups (e.g., interprofessional health care team).^25^ The key to DPI lies in providing IPE events throughout the curriculum for students across disciplines to learn together, from each other, and about each other in a healthy IPE environment that is free of bias and prejudice.^9,11^ In this way, DPI serves to reduce intergroup bias by creating a higher level ingroup identity.

Unfortunately, maintaining the current status quo of siloed education results in graduating health care professionals who have a strong uniprofessional identity with little or no recognition of belonging to an interprofessional collaborative health care team. Uniprofessional identity is automatically developed as students learn their discipline-specific values, culture, norms, and perspective of health and health care in siloed educational curricula and clinical experiences.^24^

By design, the IPE curriculum should target the reduction and/or elimination of existing stereotypes and prejudices, which if left unchecked can create a barrier that prevents successful IPCP. Once students develop a DPI, they will begin to see themselves as not only members of their own profession but also members of an interprofessional health care team.^24^ This may help students acquire the skills needed to be collaborative ready upon graduation in ways that may improve patients' health care outcomes.^4,5,9,24^

### Patient-centered care and interprofessional bias and prejudice

Over 97.5% of participants agreed with the importance of providing services that involve patient centeredness and an integration of diversity and ethics into clinical care. Yet, when asked if prejudices and assumptions get in the way of the delivery of health care, 25% of the participants reported disagreement, strong disagreement, and/or were undecided. This finding suggests that 25% of the respondents failed to recognize the impact of identified interprofessional biases among health care professionals on patient outcomes. This may stem from the lack of exposure to other health care professionals, a paucity of knowledge about their roles and responsibilities, and lack of understanding of how their profession relates to other health care disciplines and/or its impact on patient care outcomes.

A patient-centered approach requires the establishment of trust, compassion, an integration of cultural awareness and diversity, right treatment, cooperation, and an understanding of the patient's perceptions.^21–23^ Over 97% of the participants endorsed the value of patient-centered care and the importance of establishing trust with their patients. Yet, 25% of the respondents reported that interprofessional prejudices and biases are not a barrier to patient-centered care indicating a failure to recognize that patient-centered care cannot be fully achieved when coexisting with persistent interprofessional biases and prejudices among members of the health care team.

## Limitations

There are several limitations to this study. First, this study utilized a one-group post-test-only design. The coauthors experienced barriers for student participation, including scheduling and other logistical issues, which limited participation to two disciplines. The authors recommend repeating this study using a pretest/post-test control group design and including additional disciplines that are typically represented on a PC team. Due to the small sample size, generalization of findings may be limited.

## Conclusion

This study contributed to participants' knowledge of PC, and increased their understanding of the value of IPE and IPCP. In addition, the study identified the existence of interprofessional biases and prejudices that may impede IPCP resulting in reduced health care outcomes. This persistent phenomenon can be attributed, in part, to the historic academic and curricular silos in educating health care professions' students, resulting in health care graduates with uniprofessional identities. Instead, faculty and health educators are encouraged to embed IPE into curricula across disciplines to dismantle preexisting interprofessional biases and stereotypes while constructing DPI. This can be accomplished by strategically integrating interprofessional student teams using multiple teaching pedagogies (e.g., team- and case-based learning, interprofessional simulation education, and interprofessional community-based clinical experiences) for both didactic and clinical experiences, which will serve to build DPI and graduate collaborative ready professionals.^11^ IPE experiences will serve to modify student attitudes, eliminate biases, and clarify inaccurate assumptions about other health care disciplines.^24–26^ This approach aligns with Kirkpatrick's Expanded Typology Level 2a.^5^

PC could possibly serve as the perfect milieu to implement IPE in multiple health care disciplines, especially given that the competencies of PC align with IPE/IPCP competencies.^13,14^ PC requires a cohesive interprofessional team with effective communication skills, exemplary ethical standards, and a commitment to patient- and family-centered care. This approach requires well-implemented interventions, with holistic services that integrate the physical, social, spiritual, and environmental needs of the patient and family. Interprofessional holistic care distinguishes palliative services from traditional, siloed health care services.

The authors' recommendations for future IPE activities include the following: (1) explore ways that educators can help students become aware of their existing interprofessional biases; (2) model and train students to act in a supportive manner toward other health care disciplines; (3) create IPE opportunities to apply collaborative practice skills (communication, teamwork, ethics, and roles/responsibilities) in simulation and clinical settings; and (4) design more inclusive IPE PC activities to explore the role of other constituencies such as family members, social work, and physical therapy.

## Abbreviations Used

DPI: dual-professional identity
IOM: Institute of Medicine
IPAS: Interprofessional Attitudes Survey
IPCP: Interprofessional Collaborative Practice
IPE: interprofessional education
IPEC: Interprofessional Education Collaborative
JCAHO: the Joint Commission
PC: palliative care
SLP: speech-language pathology
